# Biomechanical superiority of novel dynamic tape over standard tape suture in distal triceps tendon repair: a human cadaveric study testing an intense early rehabilitation protocol

**DOI:** 10.1007/s00402-025-06040-2

**Published:** 2025-09-10

**Authors:** Moritz Kraus, Bogdan Bocea, Nicolas Ion, Mehar Dhillon, Ivan Zderic, Luise Puls, Boyko Gueorguiev, R Geoff Richards, Hans-Christoph Pape, Tatjana Pastor, Torsten Pastor

**Affiliations:** 1 AO Research Institute Davos, Davos, Switzerland; 2 Department of Trauma Surgery, University Hospital Zurich, Zurich, Switzerland; 3 Lucian Blaga University of Sibiu, Sibiu, Romania; 4 University Hospital of Basel, Basel, Switzerland; 5 Medical Faculty, University of Zurich (UZH), Zurich, Switzerland; 6 Department of Orthopaedics and Traumatology, Bürgerspital Solothurn, Solothurn, Switzerland; 7 Department for Plastic and Hand Surgery, Inselspital University Hospital Bern, University of Bern, Bern, Switzerland; 8 Department of Orthopedic and Trauma Surgery, Cantonal Hospital Lucerne,, Lucerne, Switzerland

**Keywords:** Distal triceps tendon rupture, SutureTape, DYNATape, Biomechanical assessment, Cadaveric study, Early rehabilitation

## Abstract

**Background:**

Distal triceps tendon rupture is related to high complication rates with up to 25% failures. Elbow stiffness is another severe complication, as the traditional approach considers prolonged immobilization to ensure tendon healing. Recently, a dynamic tape was designed, implementing a silicone-infused core for braid shortening and preventing repair elongation during mobilization, thus maintaining constant tissue approximation. The aim of this study was to compare biomechanically the novel dynamic tape versus conventional tape in a human cadaveric distal triceps tendon repair model.

**Methods:**

Sixteen paired arms from eight donors were split to two groups. Distal triceps tendon tenotomies and repairs were performed via the crossed transosseous locking Krackow stitch technique for anatomic footprint repair. Either conventional (SutureTape) or the novel dynamic tape (DYNATape) were used. A postoperative protocol mimicking intense early rehabilitation was simulated by a 9-day, 300-cycle daily mobilization under 150 N load followed by a final destructive test.

**Results:**

Significant differences were identified between the groups regarding the displacement over time at the distal, intermediate, and proximal tendon aspects, *p* < 0.001. DYNATape demonstrated significantly less displacement compared to SutureTape (4.6 ± 1.2 mm versus 7.8 ± 2.1 mm) and higher load to failure (637 ± 113 N versus 341 ± 230 N), *p* ≤ 0.037. DYNATape retracted 0.95 ± 1.95 mm after each 24-hour period and withstood the whole cyclic loading sequence without failure. In contrast, SutureTape failed early in three specimens.

**Conclusion:**

DYNATape demonstrated improved biomechanical competence compared to SutureTape in a distal triceps tendon repair model, with significantly lower maximal displacement and higher load to failure. These findings indicate that DYNATape may offer a more stable construct under controlled laboratory conditions. Knot slippage and bone-related complications observed in both groups underscore the technical challenges associated with this repair technique and highlight the importance of precise surgical execution.

## Introduction

Distal triceps tendon rupture is an uncommon injury in orthopedic practice. It is most often caused by direct impact to the elbow, lifting weights, or repetitive strain [1]. Despite its rarity, it poses a significant challenge due to a high complication rate [2]. Surgical repair is recommended for active individuals with complete ruptures, as well as for incomplete tears associated with loss of strength [3]. Up to 14% of patients experience a re-tear after primary repair [2]. A particularly severe complication is elbow stiffness [4], often worsened by the traditional treatment approach, involving a period of prolonged immobilization for several weeks [5, 6]. Range of motion is restricted to ensure the adequate healing and integrity of the repaired tendon [7]. An early mobilization strategy poses a risk of compromised tendon attachment to the bone [8], a critical aspect in the recovery process. In this context, the development of the DYNA-Technology ^®^ (Johnson&Johnson MedTech, Raynham, MA, USA) with an innovative suture material, has emerged as a promising path for improving surgical outcomes. This suture material, characterized by a silicone-infused core, has been designed to maintain constant tissue approximation [7]. This feature enables the suture to shorten its braid and maintaining contact pressure [9], effectively preventing repair elongation during mobilization [10]. Such advancements hold the potential to address the limitations of current surgical techniques, particularly to reduce early gapping in tendon repair.

Prior studies on DYNA-Technology laid the groundwork for this study. Biomechanical evaluations of DYNACORD^®^ sutures [7] (Johnson&Johnson MedTech, Raynham, MA, USA), a product analogous to the novel high-strength self-tightening tape DYNATape of the same manufacturer, have revealed significant benefits. A significant 27% reduction in tendon cut-through after 1000 cycles, in comparison to FiberWire^®^ sutures (Arthrex, Munic, Germany) was reported [10].

This study aimed to assess the biomechanical capacity of the novel high-strength self-tightening tape and a standard tape by employing a human cadaveric model using a crossed transosseous locking Krackow stitch technique for anatomic footprint repair [11] of the distal triceps tendon, replicating intense early rehabilitation conditions. It was hypothesized that the use of the novel high-strength self-tightening tape results in superior biomechanical performance, including reduced displacement and increased load to failure.

## Methods

Sixteen fresh-frozen human cadaveric arms were used. This study was approved by the institutional internal review board based on the approval of the specimens' delivery by Science Care Ethics Committee. The cadavers were paired arms of 8 donors (4 male, 4 female) and were utilized with each group comprising 4 left and 4 right arms randomly assigned to one of two groups (DT and ST). The age of the donors was 81.7 ± 7.6 (mean value ± standard deviation) years, and the body mass index (BMI) was 25.9 ± 9.1 kg/mm^2^. All donors gave their informed consent inherent within the donation of the anatomical gift state-ment during their lifetime.

All specimens were stored fresh-frozen at -20 °C and thawed at room temperature for 24 h prior to preparation and biomechanical testing. Distal triceps tenotomies were performed and anatomic footprint repair was performed using either a conventional (SutureTape^®^, ST, Arthrex, Munich, Germany) or the novel dynamic high-strength self-tightening tape (DYNATape^®^, DT, Johnson&Johnson MedTech, Zuchwil, Switzerland).

### Sample size calculation

To detect a mean difference in tendon displacement of 1.5 mm with a standard deviation of 1 mm, the calculated sample size indicated the necessity for approximately 7 specimens per group to achieve an 80% power at a 5% significance level. To enhance the robustness of the study and account for potential variability, this number was increased to 8 specimens per group.

### Surgical technique

**Fig. 1:**
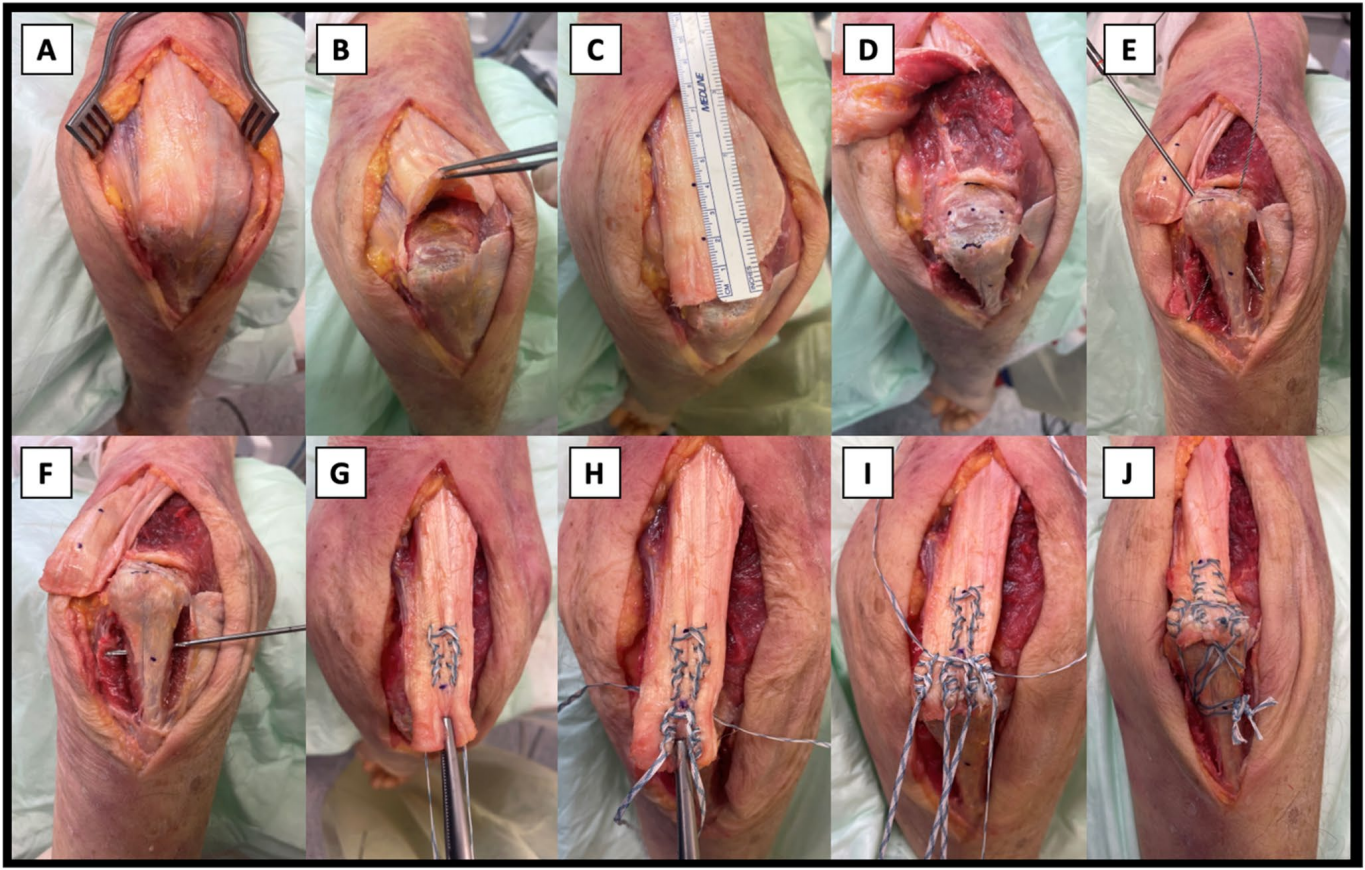
Surgical technique used in the current study. Sequential steps are involved in the surgical repair of the triceps tendon. **A** Illustrates the exposure of the triceps tendon following skin incision. **B** Depicts the tenotomy of the triceps tendon and the preparation of the tendon footprint. **C** Demonstrates the placement of marking points at 2 cm and 4 cm proximal to the distal end of the tendon. **D** Shows the marking of the entry points for the drill holes. **E** The creation of crossed drill holes is presented. **F** Visualizes the drilling of the distal horizontal hole. **G**–**I** Display the suturing of three tapes using locking Krackow stitches. **J** Exhibits the completed footprint repair of the distal triceps tendon, with knots tied securely under the anconeus muscle

For the surgical technique employed in this study, thorough steps were followed to ensure optimal outcomes. All tendon repairs were conducted by the same surgical team on the same day using the transosseus cruciate footprint repair technique as described by Anderson et al. [11] A skin incision was made centered on the junction of the middle and distal thirds of the humeral shaft, avoiding an incision over the tip of the olecranon (Fig. 1A). The distal triceps was exposed and a triceps tenotomy was performed at the footprint of the triceps (Fig. 1B). In order to handle the tendon suture at standardized intervals, markings were made 2 cm and 4 cm proximal to the distal end of the tendon with a surgical pen (Fig. 1C). Subsequently, the anconeus and flexor carpi ulnaris insertions were elevated off their respective sides on the ulna to facilitate accurate tunnel placement in a center-center position of the radial and ulnar footprint side (Fig. 1D).

Two transosseous tunnels were drilled in a cruciate order on the anteromedial and anterolateral aspects of the footprint using a 2.4-mm drill bit (Fig. 1E). Proximally these drill channels were started at a point exactly 0.5 cm radially and ulnar in the middle between the tip of the olecranon and the most proximal point of the olecranon. By aiming to a marker placed 3 cm distal to the tip of the olecranon, drilling was consequently performed at an angle of approximately 45°. Care was taken to drill through the second cortex exactly medially and laterally to this marker in the anteroposterior direction leaving an anterior bone bridge of 5 mm. Additionally, a third transverse drill tunnel was drilled from ulnar to radial connecting the exits of the previously drilled tunnels (Fig. 1F).

**Fig. 2:**
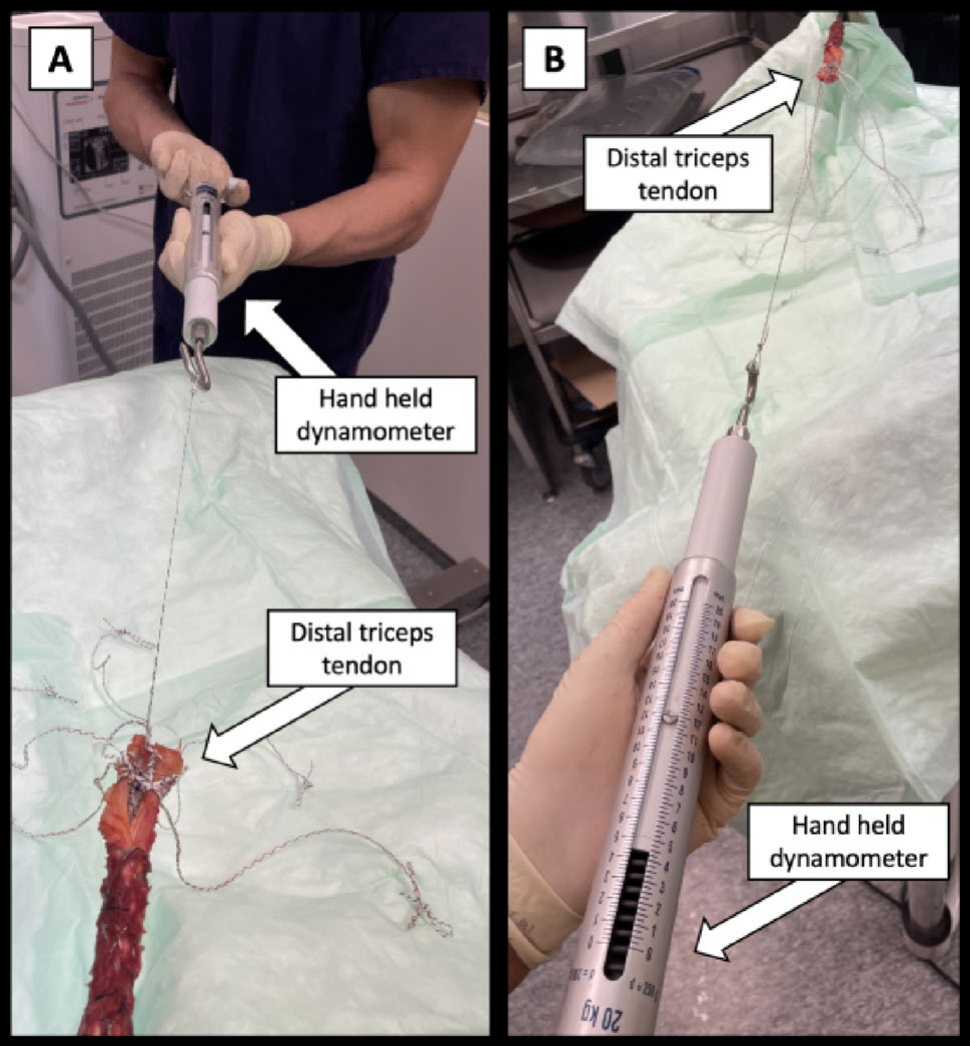
**A**, ** B** Pre-conditioning process of the tendon suture threads from two angles. Pre-conditioning involved applying a longitudinal force to each suture along the tendon with 120 N for 10 s

Three tape sutures (size #2), employing a locking Krackow technique with 5 stitches on each side were used. The first tape (I) was sewed centrally into the distal 4 cm of the tendon, exiting the tendon 2 cm proximal of its distal end on the humeral side of the tendon (Fig. 1G). The distance between the sutures exiting the anterior aspect of the tendon was matched to the distance between the anteromedial and anterolateral transosseous drill tunnels. A second locking Krackow suture tape (II) was positioned, exiting the posterior and distal aspect of the tendon at its peripheral medial and lateral margins (Fig. 1H). Similarly, a third suture tape (III) was placed in the medial and lateral portion of the tendon, following the method of the second placement (Fig. 1I). This third suture tape was marked with a surgical pen for later identification. The assistant aided with suture guidance as well as constant suture traction to ensure optimal stitches and coverage of the tendon footprint on the olecranon to minimize gap formation during testing as previously reported [12, 13]. Prior to knot tying, each suture end was individually preconditioned for 10 s using a manually held dynamometer with 120 N along the direction of traction (Fig. 2), in order to optimally secure the Krakow locking suture. The sutures exiting the humeral aspect of the tendon (I) were passed through their respective drill tunnels in the footprint using a suture passer [11]. The lateral limb of the centrally placed suture (II) and the medial limb of the peripherally placed suture (III) were shuttled through the transverse drill tunnel from medial to lateral. Conversely, the medial limb of the centrally placed suture (II) and the lateral limb of the peripherally placed suture (III) were shuttled from lateral to medial.

The suture exiting the anterior aspect of the tendon (I) was tied first with the elbow in full extension. The knot was positioned on the radial side of the olecranon, deep to the anconeus muscle. The peripheral posterior suture (III) was tied in a similar fashion on the radial side of the olecranon. Finally, the central posterior suture (II) was passed through the triceps tendon in two points above the footprint and tied down to complete the repair. Tying this suture proximally served to increase compression at the footprint while reducing knot bulkiness distally (Fig. 1J). All knots were tied with 7 alternating surgeon's knots, which according to Neuhofer et al. [14] as well as Pastor et al. [15], achieves a secure knot configuration in high-strength sutures exposed to high mechanical loads [16]. After completing the distal triceps tendon repair, a monocortical reference hole was drilled 2 mm below the horizontal drill channel. Suture markers (2 − 0 Vicryl, Johnson&Johnson MedTech, Rariton, NJ, USA) were placed in the tendon distal to the repair (distal), in the middle between suture I and II (intermediate) and directly proximal to the suture I (proximal) to denote measurement points along the repair construct. The cranial edge of the reference hole was used as the starting point for posterior distance measurements extending to the three different suture markers, defining the distal, intermediate and proximal measurement.

### Biomechanical testing

**Fig. 3:**
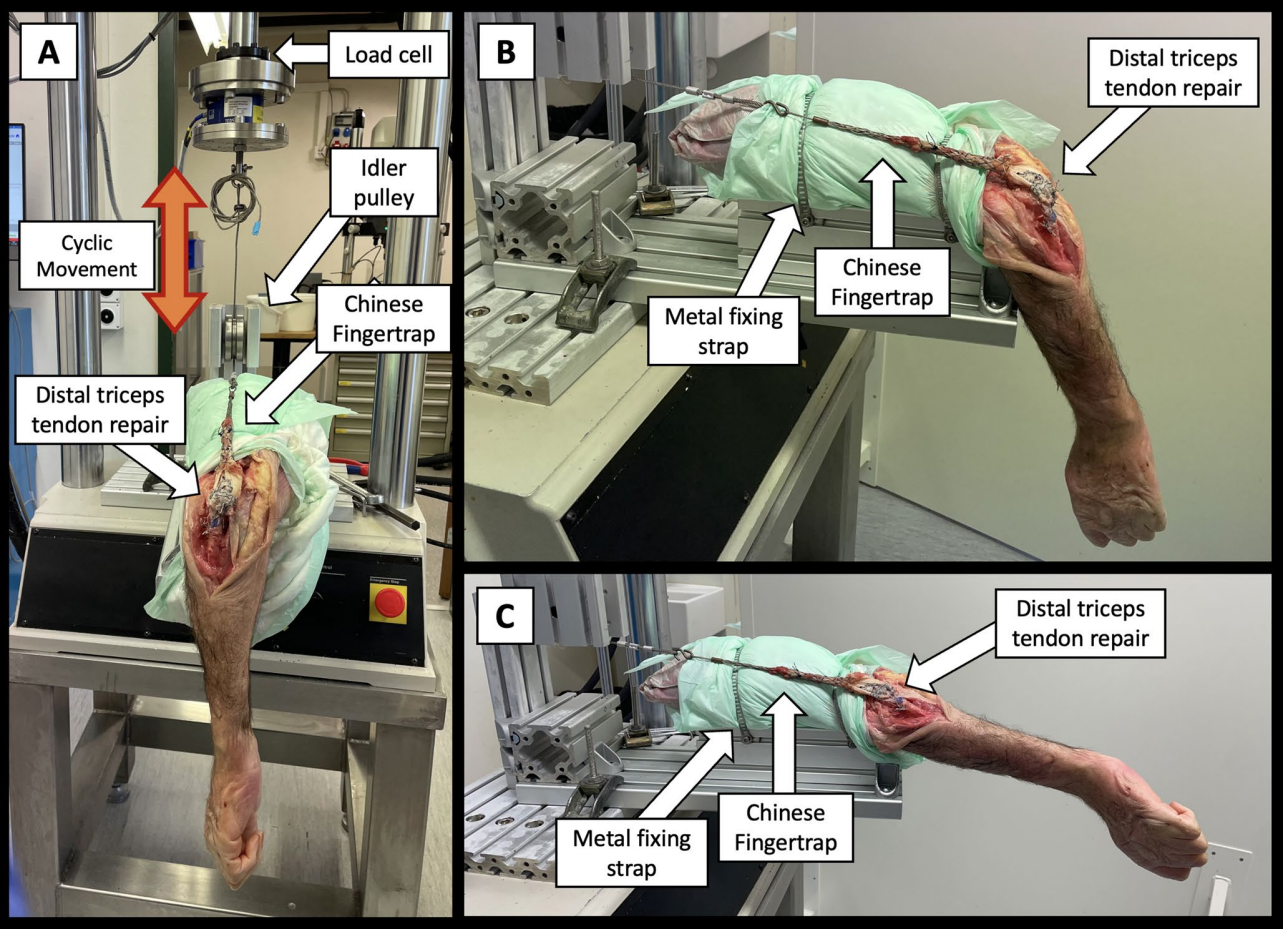
**A** Test setup with a right specimen mounted for biomechanical testing in 90° flexion. **B** View from lateral. **C **Fully extended elbow

A servo hydraulic material testing machine (MTS MiniBionix II, MTS Systems Corp., Eden Prairie, MN, USA) equipped with a 5 kN load cell was used for biomechanical testing. The specimens were fixed in a retaining form that provided stable anchoring of the humerus by means of two tensionable 0.5 cm wide metal bands, in line with the pulling direction of the testing machine with the elbow flexed to 90° and the forearm vertically exactly at the end of the testing table (Fig. 3). The triceps tendon was secured through a finger trap construct starting 4 cm proximal to the sutures of the triceps repair and attached to the load cell via a steel cable and an idler pulley.

### Test protocol

The testing protocol included a two-day immobilization at 6 °C, followed by a 9-day, 300-cycle daily mobilization regimen, simulating an intense early rehabilitation protocol against gravity. The testing protocol of each day included a preconditioning phase of 10 cycles at 0.25 Hz and 120 N, followed by 300 cycles at 0.25 Hz of a sinusoidal loading protocol with a peak load of 150 N to simulate physiologic elbow extension against gravity from 90° flexion (Fig. 3B) to complete elbow extension (Fig. 3C). The loading protocol, based on a previously published model [17], simulating an early postoperative rehabilitation through 300 daily cycles represented a typical 25-minute physical therapy session over nine days reflecting a standard rehabilitation series in Switzerland. Peak loads of 150 N were determined during pilot testing to reach full extension to reflect physiologic movement. This was followed by a test to catastrophic failure. Catastrophic failure testing was conducted with the forearm fixed horizontal (Fig. 2C) and the machine pulling axial over a pulley at a rate of 0.1 mm per second till catastrophic failure defined as a sudden decrease in the load in the load displacement curve. Primary failure mechanisms include suture cut-out from tendon, bone, knot unraveling, complete olecranon fracture, elbow dislocation, or complete unraveling of knots.

### Measurements and data acquisition

Before and after each test, the distance from the reference hole to the distal, intermediate, and proximal suture markers, were measured. We measured the distance from the radial and ulnar footprints to where the suture entered the tendon from the radial and ulnar footprints. Shortening of the tendon-bone construct during the rest period — defined as the approximately 24-hour interval between loading sessions under refrigerated, moist conditions — was assessed by measuring the change in construct length. This change between the pre-measurement and the post-measurement from the previous day was calculated and is reported as tendon repair length variation. All measurements were taken by two independent investigators separately using a highly accurate measuring gauge with an accuracy of 0.02 mm (Futuro, Brütsch/Rüegger, Urdorf, Switzerland) with the arm placed outstretched on a side table and under 10 N load to the triceps tendon using a dynamometer in parallel to the humerus axis, to achieve reproducible measurement conditions. A displacement of the triceps relative to its footprint of 10 mm proximal to its original position on the footprint was defined as clinically relevant failure criterion as this leaves the footprint completely uncovered.

Machine data in terms of load and displacement were recorded from the machine controllers at 100 Hz. Based on these data, peak load before catastrophic failure was extracted from the load-displacement curve. Catastrophic failure was defined as a sudden decrease in the load-displacement curve, representing the transition from elastic to plastic deformation.

### Statistical analysis

Data were analyzed using Shapiro-Wilk and Levene’s tests for normality and homogeneity of variances, Paired-Samples *t*-tests for within-subject comparisons, and General Linear Model Repeated Measures for between-subject comparisons.

A repeated-measures Analysis of Variance (ANOVA) to analyze data from the five anatomical measurement sites was employed. The primary focus was to examine the effects of two factors: ‘Group’ and ‘Time’, as well as their interaction, on these measurements. ‘Group’ represented different sutures while ‘Time’ referred to the longitudinal aspect of the measurements. Specimens were measured multiple times across a defined period, allowing for the assessment of within-subject changes. The ANOVA was structured to distinguish between-subjects effects (focusing on ‘Group’) and within-subjects effects (focusing on ‘Time’ and the interaction between ‘Group’ and ‘Time’). This approach provides insights into how measurements changed over time and whether these changes differed across groups.

To ensure manual measurement accuracy, the Intraclass Correlation Coefficient (ICC) was calculated for all measurement sites, to quantify the agreement among raters, specifically using the ICC (A,1) model which is designed for single measurement units and focuses on the type of agreement. This analysis was conducted through the “icc()” function within the “irr” package in R. The ICC values were interpreted based on Cicchetti’s guidelines [18], categorizing agreement as poor (< 0.40), fair (0.40–0.59), good (0.60–0.74), or excellent (0.75-1.00). To enhance the robustness of our findings, 95% confidence intervals (CI) for each ICC value were calculated as previously advised [19].

## Results

### Tendon repair length variation during rest period

Analysis of the mean reduction in measurements during the rest period, as summarized in Table 1, revealed significant differences between groups at every location of measurement. In the distal location, the change for ST was 0.644 ± 0.731 mm, whereas DT exhibited a more pronounced reduction of -1.354 ± 1.024 mm (*p* < 0.001). At the intermediate location, ST showed a change of -0.008 ± 0.551 mm in contrast to the DT group which had a reduction of -2.030 ± 1.09 mm (*p* < 0.001). The proximal measurements followed a similar trend; ST had a change of 0.238 ± 1.210 mm while DT showed a reduction of -2.162 ± 1.088 mm (*p* < 0.001). (Fig. 5B).

**Table 1:** Change of measured tendon repair length in terms of mean value and standard deviation (SD) during the rest period by location (distal, intermediate, proximal) and group (SutureTape (ST); DYNAtape (DT))

Measurement	Group	Mean tendon repair length variation [mm]	SD [mm]
Distal	SutureTape	0.644	0.731
	DYNATape	-1.354	1.024
Intermediate	SutureTape	0.008	0.551
	DYNATape	-2.030	1.092
Proximal	SutureTape	0.238	1.210
	DYNATape	-2.162	1.088

Negative values indicate a decrease in measurement during the rest period, while positive values indicate an increase

**Fig. 4:**
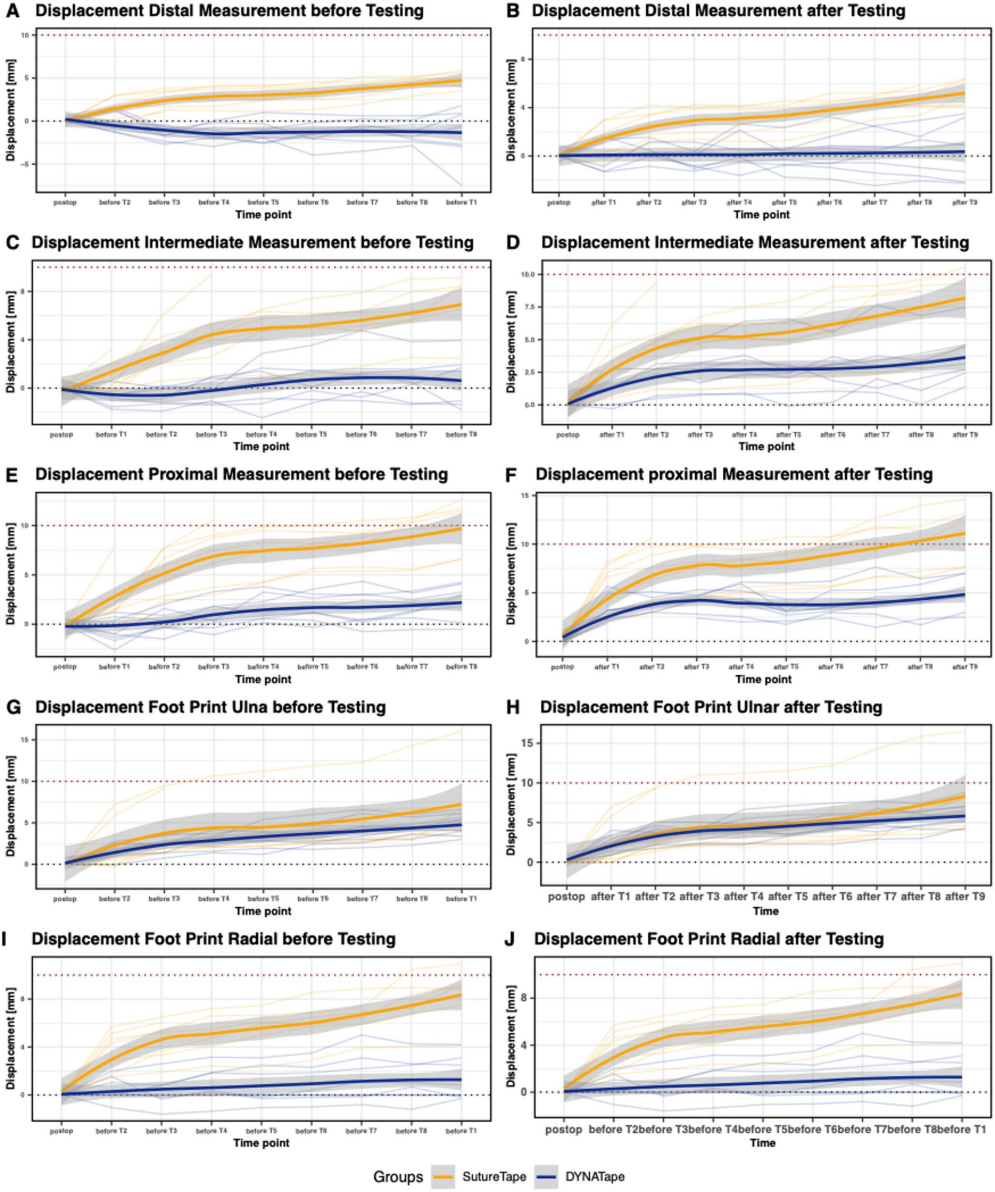
Longitudinal displacement measurements for DT and ST before and after testing. This multi-panel figure presents longitudinal displacement data for the groups DT (blue) and ST (orange), across various anatomical locations and time points, both before and after testing. Panels **A**, **C**, **E**, **G**, and **I** illustrate the displacement measurements before testing, while Panels **B**, **D**, **F**, **H**, and **J** show the measurements after testing for distal, intermediate, proximal, ulnar footprint, and radial footprint locations, respectively. Each line represents the mean displacement trajectory for the group over time, with shaded areas indicating the 95% confidence intervals. The dotted red lines represent the arbitrary defined clinically relevant failure of 10 mm

Before testing, (Fig. 4), panels A, C, E, G, and I demonstrate that both groups exhibited similar trends of displacement across all anatomical locations, with DT consistently demonstrating slightly lower displacement levels than ST. After testing, (Fig. 4), panels B, D, F, H, and J indicate a noticeable divergence between the two groups. The DT group maintained lower displacement measurements compared to the ST group at the distal, intermediate, and proximal locations, as well as the ulnar and radial footprints. This trend was consistent across all time points post-testing, with DT showing a superior ability to resist displacement.

Repeated measures ANOVA results for each measurement location are summarized in Table 2. In the distal, intermediate, and proximal measurements, ‘Group’ had a highly significant effect (p < 0.01), while ‘Time’ alone was not significant. Significant within-subject effects for ‘Time’ and ‘Group × Time’ interaction (*p* < 0.001) were observed at all sites. This indicates that the change in distance differed between groups and evolved differently over time, while Time alone showed no significant main effect across subjects.

**Table 2:** Detailed results of repeated measures ANOVA

Measurement Location	Effect of group between subjects (*p*-value)	Effect of time between subjects (*p*-value)	Effect of time within subjects (*p*-value)	Interaction group × time (*p*-value)
Distal	0.0012	0.646	< 0.001	< 0.001
Intermediate	0.0016	0.924	< 0.001	< 0.001
Proximal	< 0.001	0.653	< 0.001	< 0.001
Ulnar Footprint	0.532	0.985	< 0.001	< 0.001
Radial Footprint	< 0.001	0.369	< 0.001	< 0.001

### Footprint tendon displacement during testing and load to failure

DT demonstrated a significantly lower maximal displacement, averaging all displacement measurement locations to 4.6 ± 1.2 mm, compared to 7.8 ± 2.1 mm observed with ST (*p* = 0.037).

**Fig. 5:**
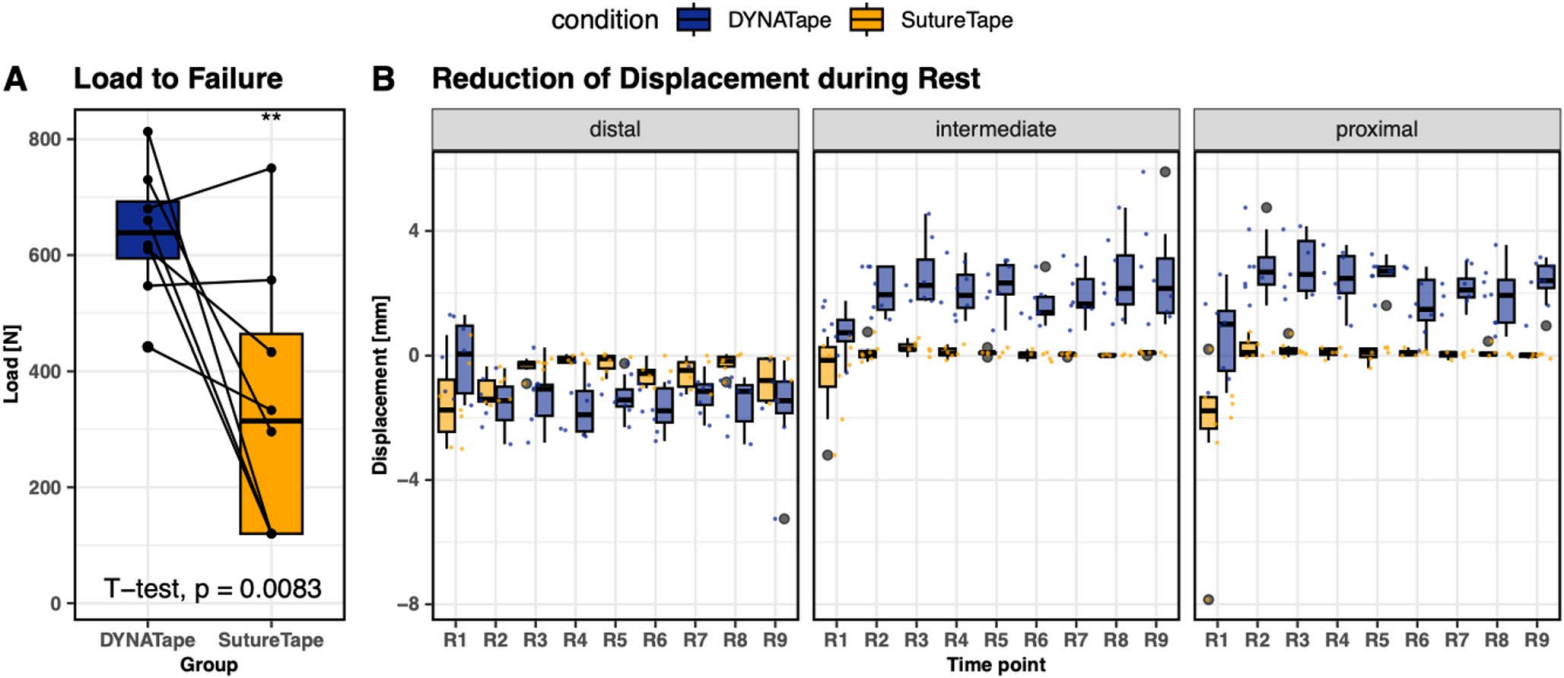
Comparative analysis of load to failure and reduction of displacement during rest period between DT and ST. **A** Load to failure for both DT (blue) and ST (orange), with individual paired measurements connected by lines and summarized in boxplots. The statistical significance is indicated by asterisks, with the *p*-value provided from a *t*-test. **B** Depicts the reduction of displacement during rest period across nine consecutive rest intervals (R1 to R9) for both groups at distal, intermediate, and proximal measurement locations. Each boxplot represents the distribution of measurements, with the central line indicating the median, the box boundaries representing the interquartile range, and the whiskers extending to the most extreme data points not considered outliers

In terms of load to failure, DT outperformed ST with a higher load of 637 ± 113 N, compared to 341 ± 230 N for ST; *p* = 0.008. (Fig. 5A).

### Failure modes

**Fig. 6:**
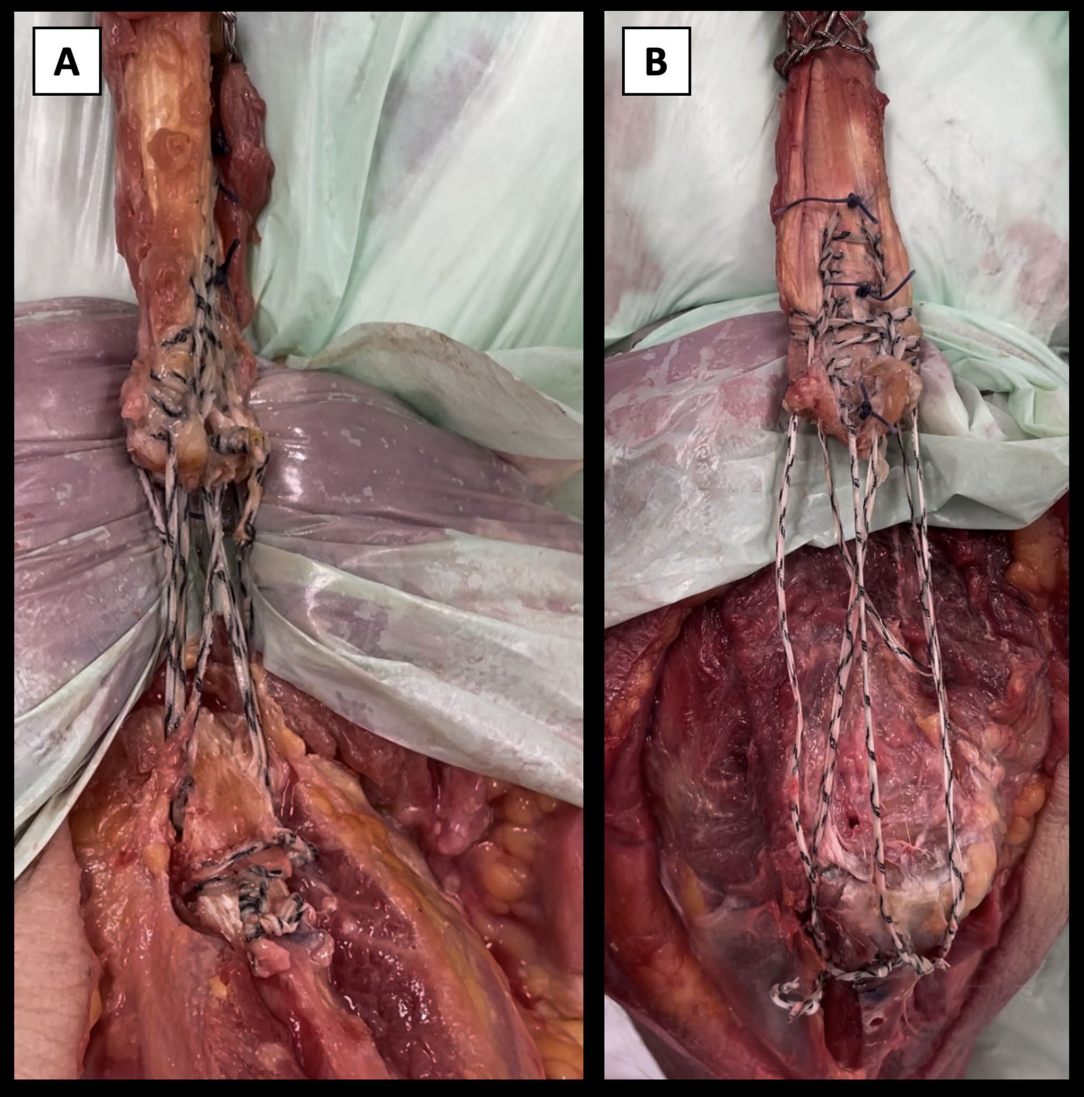
Failure modes: **A** cut through bone, **B** suture unrevealing as failure

Throughout cyclic loading, DT withstood the entire process without catastrophic or clinically relevant failure. In contrast, ST exhibited early catastrophic failure through knot unraveling in three instances, occurring after 256, 850, and 2410 cycles. All specimens were included in the analysis without exclusion of outliers. Both groups experienced similar modes of failure during the final failure test, with one tendon cut-out and one cut through the bone. Additionally, there was a total of 4 instances of knot unraveling (Fig. 6B) with DT and 5 with ST, indicating a comparable challenge in maintaining knot integrity under stress especially after cyclic loading. In terms of catastrophic failure, two specimens in the DT group sustained olecranon fractures (Fig. 6A), while one specimen in the ST group experienced posterolateral elbow dislocation.

### Interrater reliability

The interrater reliability analysis revealed a high degree of consistency among the two raters across most of the measured locations. The ICC for the distal measurement was 0.995 (CI: 0.994–0.997; *p* < 0.001), for the intermediate measurement it was 0.993 (CI: 0.991–0.994; *p* < 0.001), and for the proximal measurement – 0.997 (CI: 0.996–0.997; *p* < 0.001), all indicating excellent agreement, suggesting a high level of precision in measurements taken at these anatomical sites. For the ulnar footprint measurement the ICC was 0.724 (CI: 0.658–0.778; *p* < 0.001), denoting good agreement, which, while being lower than at the other sites, still indicates a reliable level of consistency among raters. The radial footprint measurement also showed excellent agreement with an ICC of 0.99 (CI: 0.986–0.992; *p* < 0.001).

## Discussion

This study investigates the biomechanical performance of the novel DT and the conventional ST for distal triceps tendon repair, demonstrating reduced displacement and superior load to failure for DT compared to ST. These characteristics could significantly improve the DT repair stability and support more progressive early rehabilitation strategies, potentially optimizing patient recovery outcomes.

Comparative evidence from Achilles tendon repair studies shows that early functional rehabilitation does not compromise repair integrity, boosts patient satisfaction and expedites return to normal activities [20]. This is even more relevant to triceps tendon repair, where early rehabilitation can significantly reduce the incidence of elbow stiffness.

DT`s retraction capacity, coupled with its durability under cyclic loading, reflects a material characteristic that enhances repair strength. The tape’s ability to withstand the intense cyclic load protocol is essential to enable faster rehabilitation following tendon repairs.

The observed failures, primarily through suture unraveling, raise important considerations regarding surgical technique and the inherent properties of the sutures. While both suture types exhibited this mode of failure, the higher incidence in ST suggests a potential material limitation. In this context the results of the current study are in conflict with previous published reports investigating the security of alternating surgeons’ knots. Neuhofer et al. stated, that 7 knots are necessary to achieve 100% knot security in conventional round high strength sutures which was later confirmed by van Knegsel et al. in different ambient conditions [14, 16]. However, both studies used a monotonic quasi-static ramp until failure. The results of the current study implicate that the number of 7 knots might not be transferable to cyclic loading conditions in a more realistic setting, which should be evaluated in future biomechanical research. The occurrence of bone-related complications, such as olecranon fractures with DT and elbow dislocation with ST, highlights the strength of both suture materials, as they are stronger than the patient’s bone in some cases.

From a clinical perspective, increased re-rupture and re-operation rates are associated with transosseous fixation, then achieved through anchor fixation [21]. Our research complements these insights, demonstrating that DT significantly reduces displacement and enhances load capacity. This suggests a potential reduction of early gapping, akin to the benefits observed with anchor fixation [22]. 

A biomechanical study investigating anchor repair [23] reported a load to failure of 510.5 ± 67.6 N and a displacement of 2.67 ± 1.08 mm [22]. In the present study, DT transosseous repair demonstrated higher load to failure and lower displacement after extensive cyclic loading. Therefore, it would be interesting for future studies to see whether the combination of DT and knotless bone anchor shows even better biomechanical performance. This surgical technique might offer the possibility for adequate anatomic footprint repair. Compared to cruciate repair and anchor repair, it shows the best anatomic footprint coverage and least displacement under cyclic load [24]. Yet, it must be admitted, that a recent review of 184 cases [25] favors anchor repair over transosseus repair, as a significant higher failure and revision rate occurred with transosseus repair compared to anchor repair [25]. Nevertheless, we decided to perform a transosseous triceps repair in the current study as the two different tapes are only available on two different anchor systems, which could have biased the results.

The biomechanical characteristics of the cruciate anatomic footprint repair in this study can be contrasted with previous studies that compared triceps displacement between transosseous cruciate and suture anchor repair groups [26], where no significant differences were observed at 1500 cycles. The lack of disparity in performance between the transosseous cruciate and suture anchor repair techniques suggested that both methods provided comparable biomechanical integrity when an equal number of sutures were used [26].

The displacement of ST in the current study is consistent with previously published data [8, 27, 28]. It is noteworthy that the use of DT resulted in significantly reduced displacement, despite being observed over double the number of loading cycles in this study compared to the prior standard [27].

DT’s robust biomechanical qualities might allow for earlier movement post-surgery while maintaining repair integrity, potentially lowering the incidence of post-surgical elbow stiffness, which detrimentally affects patient recovery.

Knot slippage with complete unravelling of the knot was the primary failure mechanism observed, raising concerns about knot security. Although prior studies show that suture tapes have adequate loop security under cyclic loading [29], the higher load and increased number of cycles during our testing, along with the inclusion of physiological movement simulation, may account for the observed knot slippage with suture unravelling. Furthermore, biomechanical research revealed an improved knot security for DT which in contrast to ST only needs 4 knots in order to achieve a secure knot instead of 7 knots needed in ST [15]. Nevertheless, we decided to use 7 knots in both groups in order to evaluate both tapes in a standardized manner. It is likely that similar results would have been achieved if only 4 knots were used in the DT group, leading to smaller knot stacks and, therefore, less hardware irritation.

Additional insights indicate that the DYNACORD Suture, with four half hitches, matches the knot security offered by the FiberWire^®^ suture with seven half hitches [16]. The DYNA’s knot security is comparable but with fewer knots, attributed to the hydration and expansion of the silicone/NaCl core implemented in the DYNA-technology [30], resulting in a tighter knot and a small knot stack. Validating these results in anatomic settings is crucial due to potential benefits such as improved knot and re-tensioning security, smaller knot stacks, and reduced patient discomfort.

Both the ST and DT suture materials remained intact during our tests, indicating that they are more stable than the tissue they are intended to fix. The healing of the sutured tendon depends on the proper adaptation of the tissue and the maximum possible contact pressure of the tendon to the physiological tendon footprint [31]. DT was more effective in reducing overall dislocation of the tendon from the footprint. The DT‘s competence also led to a reduction in dislocation during the night, which is likely to improve tendon healing and accelerate rehabilitation. This could ultimately reduce missed work time and shorten the time needed to return to sports. Particularly when critically interpreting the displacement found on the ulnar footprint, it must be taken into account that this study was designed as a worst-case scenario, where patients are allowed to actively extend their elbows on postoperative day 3 using an aggressive active motion protocol against gravity. It was hypothesized that a more moderate rehabilitation protocol, such as allowing only passive and active motion without gravity, can reduce stress on the surgical site while preventing postoperative elbow stiffness. Moreover, in a real-world setting, the forearm muscles that stabilize the elbow joint could reduce the isolated load on the triceps tendon. Nevertheless, future clinical research is necessary to transfer these promising biomechanical findings into improved patient care.

### Limitations

The biomechanical tests were conducted on elderly cadaveric specimens, with a mean donor age over 80 years. The advanced age of these specimens may limit the generalizability of our findings to younger, more active individuals. The predominance of bone-related failures—such as olecranon fractures—could reflect compromised bone quality. We employed a transosseous repair technique to standardize the fixation method across both tape types. This choice may limit the direct clinical translatability of the findings to anchor based fixation. Our experimental model lacks the biological factors involved in tendon healing, such as inflammation, vascularization, and tissue remodeling. While our nine-day cyclic loading protocol was designed to mimic early postoperative mobilization, the absence of a healing response means that our results reflect initial mechanical performance rather than long-term repair integrity.

The uniform surgical technique, performed by one team, may not reflect the outcome variability seen in broader clinical practice. Additionally, the visual measurement method, despite lacking more precise strain instruments and relying on a gauge to measure distances between suture marks and a reference hole, showed excellent interrater reliability, suggesting the method’s consistency and precision. However, the study’s scope was limited to biomechanical performance, not encompassing biological healing processes. It also did not investigate the implications of suture material on bone integrity thoroughly, as evidenced by bone complications observed, pointing to a gap in fully understanding suture-bone interactions.

Future research should aim to address these limitations, particularly by varied surgical techniques, and long-term clinical outcomes. Ultimately, the goal is to enhance patient care by providing evidence-based guidance for the surgical repair of distal triceps tendon ruptures.

## Conclusion

DYNATape demonstrated improved biomechanical competence compared to SutureTape in a distal triceps tendon repair model, with significantly lower maximal displacement and higher load to failure. These findings indicate that DYNATape may offer a more stable construct under controlled laboratory conditions. Knot slippage and bone-related complications observed in both groups underscore the technical challenges associated with this repair technique and highlight the importance of precise surgical execution.

## Data Availability

The corresponding author provides the data upon request.

## References

[1] Waterman B, Dean R, Veera SS et al (2019) Surgical repair of distal triceps tendon injuries: short-term to midterm clinical outcomes and risk factors for perioperative complications. Orthop J Sport Med 7:null. 10.1177/2325967119839998 10.1177/2325967119839998 PMC6492365 31069242

[2] David M, Macknet MD, Ford MDSE, Mak BSMARA et al (2022) Complications after traumatic distal triceps tears: an analysis of 107 cases. JSES Rev Rep Tech 2:520–525. 10.1016/j.xrrt.2022.05.004 37588465 10.1016/j.xrrt.2022.05.004 PMC10426459

[3] Yeh PC, Dodds SD, Smart RL et al (2010) Distal triceps rupture. J Am Acad Orthop Surg 18:31–40 10.5435/00124635-201001000-00005 20044490

[4] Giannicola G, Bullitta G, Rotini R et al (2018) Results of primary repair of distal triceps tendon ruptures in a general population: a multicentre study. Bone Joint J 100B:610–616. 10.1302/0301-620X.100B5.BJJ-2017-1057.R2 10.1302/0301-620X.100B5.BJJ-2017-1057.R2 29701103

[5] Phelps BM, Fomunung C, Singer W et al (2024) Postoperative rehabilitation and Return-to-Sport criteria after distal triceps rupture repair: a scoping review. Orthop J Sport Med 12. 10.1177/23259671241275956 10.1177/23259671241275956 PMC11497518 39444937

[6] Ritsch M, Regauer M, Schoch C (2022) Operative therapie der distalen trizepssehnenruptur. Oper Orthop Traumatol 34:438–446. 10.1007/s00064-022-00781-8 36094541 10.1007/s00064-022-00781-8

[7] Heygen M (2019) Evaluating the efficacy of DYNACORD™ suture

[8] Alnaji O, Erdogan S, Shanmugaraj A et al (2021) The surgical management of distal triceps tendon ruptures: a systematic review. 10.1016/j.jse.2021.06.019. J shoulder Elb Surg null:null 10.1016/j.jse.2021.06.019 34343662

[9] Horneff J, Aleem AW, Nicholson TA et al (2017) Functional outcomes of distal triceps tendon repair comparing transosseous bone tunnels with suture anchor constructs. J Shoulder Elb Surg 26 12:2213–2219. 10.1016/j.jse.2017.08.006 10.1016/j.jse.2017.08.006 29032989

[10] Owens BD, Algeri J, Liang V, DeFroda S (2019) Rotator cuff tendon tissue cut-through comparison between 2 high–tensile strength sutures. J Shoulder Elb Surg 28:1897–1902. 10.1016/j.jse.2019.02.028 10.1016/j.jse.2019.02.028 31085035

[11] Anderson CN (2020) All-Suture anatomic footprint repair of the distal triceps tendon. Arthrosc Tech 9:e2013–e2019. 10.1016/j.eats.2020.08.019 33381413 10.1016/j.eats.2020.08.019 PMC7768200

[12] Krushinski EM, Parks BG, Hinton RY (2010) Gap formation in transpatellar patellar tendon repair. Am J Sports Med 38:171–175. 10.1177/0363546509343802 19755721 10.1177/0363546509343802

[13] Jordan MC, Hoelscher-Doht S, Fehske K et al (2015) Bunnell or cross-lock Bunnell suture for tendon repair? Defining the Biomechanical role of suture pretension. J Orthop Surg Res 10:192. 10.1186/s13018-015-0331-4 26714631 10.1186/s13018-015-0331-4 PMC4696145

[14] Neuhofer S, Wieser K, Lajtai G et al (2014) Surgical knot tightening: how much pull is necessary? Knee surgery. Sport Traumatol Arthrosc 22:2849–2855. 10.1007/s00167-013-2452-9 10.1007/s00167-013-2452-9 23494026

[15] Pastor T, Zderic I, van Knegsel KP et al (2024) How many knots are necessary to achieve knot security of two high strength suture tapes? A Biomechanical comparative analysis. Arch Orthop Trauma Surg 145:43. 10.1007/s00402-024-05638-2 39680173 10.1007/s00402-024-05638-2

[16] van Knegsel KP, Zderic I, Kastner P et al (2023) Knot holding capacity of two different high-strength sutures—a biomechanical analysis. Int Orthop. 10.1007/s00264-023-06041-z 38015209 10.1007/s00264-023-06041-z

[17] Pastor T, Zderic I, Dhillon M et al (2024) New dynamic suture material for tendon transfer surgeries in the upper extremity—a biomechanical comparative analysis. Arch Orthop Trauma Surg. 10.1007/s00402-024-05322-5 38693291 10.1007/s00402-024-05322-5 PMC11211109

[18] Cicchetti DV (1994) Guidelines, criteria, and rules of thumb for evaluating normed and standardized assessment instruments in psychology. Psychol Assess 6:284–290. 10.1037/1040-3590.6.4.284

[19] Liljequist D, Elfving B, Skavberg Roaldsen K (2019) Intraclass correlation—a discussion and demonstration of basic features. PLoS ONE 14:e0219854. 10.1371/journal.pone.0219854 31329615 10.1371/journal.pone.0219854 PMC6645485

[20] Zhao J-G, Meng X-H, Liu L et al (2017) Early functional rehabilitation versus traditional immobilization for surgical Achilles tendon repair after acute rupture: a systematic review of overlapping meta-analyses. Sci Rep 7:39871. 10.1038/srep39871 28054658 10.1038/srep39871 PMC5215510

[21] Bonicoli E, Giuntoli M, Ipponi E et al (2020) Chronic tear of the distal triceps tendon treated with suture anchors and fascia Lata allograft: case report, surgical technique and literature review. Tech Shoulder Elb Surg 21:79–83. 10.1097/BTE.0000000000000197

[22] Clark J, Obopilwe E, Rizzi A et al (2014) Distal triceps knotless anatomic footprint repair is superior to transosseous cruciate repair: a biomechanical comparison. Arthrosc J Arthrosc Relat Surg 30:1254–1260. 10.1016/j.arthro.2014.07.005 10.1016/j.arthro.2014.07.005 25281349

[23] DeLong ER, DeLong DM, Clarke-Pearson DL (1988) Comparing the areas under two or more correlated receiver operating characteristic curves: a nonparametric approach. Biometrics 44:837–845 3203132

[24] Yeh P, Stephens K, Solovyova O et al (2010) The distal triceps tendon footprint and a Biomechanical analysis of 3 repair techniques. Am J Sports Med 38:1025–1033. 10.1177/0363546509358319 20200322 10.1177/0363546509358319

[25] Tran D, Yetter T, Somerson J (2022) Surgical repair of distal triceps rupture: a systematic review of outcomes and complications. JSES Rev Rep Tech 2:332–339. 10.1016/j.xrrt.2022.04.001 37588859 10.1016/j.xrrt.2022.04.001 PMC10426566

[26] Carpenter SR, Stroh DA, Melvani R et al (2018) Distal triceps transosseous cruciate versus suture anchor repair using equal constructs: a biomechanical comparison. J Shoulder Elb Surg 27 11:2052–2056. 10.1016/j.jse.2018.05.025 10.1016/j.jse.2018.05.025 30093233

[27] Scheiderer B, Imhoff F, Morikawa D et al (2018) The V-Shaped distal triceps tendon repair: a comparative biomechanical analysis. Am J Sports Med 46:1952–1958. 10.1177/0363546518771359 29763339 10.1177/0363546518771359

[28] Sequeira SB, Imbergamo C, Gould HP et al (2022) A Biomechanical comparison between transosseous cruciate sutures and suture anchors for triceps tendon repair: a systematic review and meta-analysis. Curr Orthop Pract 33:538–542. 10.1097/BCO.0000000000001162

[29] Ensminger WP, McIff T, Vopat B et al (2021) Mechanical comparison of High-Strength tape suture versus high-strength round suture. Arthrosc Sport Med Rehabil 3:e1525–e1534. 10.1016/j.asmr.2021.07.014 10.1016/j.asmr.2021.07.014 PMC8527324 34712990

[30] Bhattacharyya S, Mafilios M, Dave Spenciner PE, ME BS (2018) DYNACORD™ Suture: comparative evaluation of knot security in a mechanical model. In: DePuy Synth. https://www.jnjmedtech.com/sites/default/files/user_uploaded_assets/pdf_assets/2021-04/115006-190523-DYNACORDKnotSecurity.pdf

[31] Park JS, McGarry MH, Campbell ST et al (2015) The optimum tension for bridging sutures in transosseous-equivalent rotator cuff repair. Am J Sports Med 43:2118–2125. 10.1177/0363546515590596 26150589 10.1177/0363546515590596

